# Three-Dimensional Flow of an Oldroyd-B Nanofluid towards Stretching Surface with Heat Generation/Absorption

**DOI:** 10.1371/journal.pone.0105107

**Published:** 2014-08-29

**Authors:** Waqar Azeem Khan, Masood Khan, Rabia Malik

**Affiliations:** Department of Mathematics, Quaid-i-Azam University, Islamabad, Pakistan; Washington State University, United States of America

## Abstract

This article addresses the steady three-dimensional flow of an Oldroyd-B nanofluid over a bidirectional stretching surface with heat generation/absorption effects. Suitable similarity transformations are employed to reduce the governing partial differential equations into coupled nonlinear ordinary differential equations. These nonlinear ordinary differential equations are then solved analytically by using the homotpy analysis method (HAM). Graphically results are presented and discussed for various parameters, namely, Deborah numbers 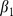 and 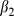, heat generation/absorption parameter 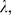 Prandtl parameter 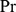, Brownian motion parameters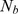, thermophoresis parameter 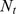 and Lewis number 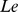. We have seen that the increasing values of the Brownian motion parameter 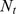 and thermophoresis parameter 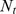 leads to an increase in the temperature field and thermal boundary layer thickness while the opposite behavior is observed for concentration field and concentration boundary layer thickness. To see the validity of the present work, the numerical results are compared with the analytical solutions obtained by Homotopy analysis method and noted an excellent agreement for the limiting cases.

## Introduction

During the past few years, study of the boundary layer flow of nanofluids over a linear stretching surface has become more and more attractive because of its numerous applications in industrial manufacturing. With regard to the sundry application of nanofluids, the researchers have been given considerable attention to improve heat transfer using nanofluids. Regular fluids, such as ethylene, water, glycol mixture and some types of oil have low heat transfer rates. Therefore it is necessary to improve some physical properties such as thermal conductivity and heat transfer rate of conventional fluids by the utilization of nanoparticles in base fluid. The term nanofluid was first time introduced by Choi [1]. In another paper, Choi et al. [2] observed that thermal conductivity of pure fluid can be increased by a factor of 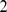 with an addition of one percent by volume fraction of the nanoparticle.

Sakiadis [3] was the first who investigated the boundary layer flow on a continuous stretching surface. In his paper, he provided numerical solutions of the boundary layer flow over a continuous stretching surface. Later on Crane [4] analyzed the exact solution of boundary layer flow of Newtonian fluid due to stretching of an elastic sheet moving linearly in its own plane. Wang [5] investigated the free convection on a vertical stretching surface. Heat transfer analysis over an exponentially stretching continuous surface was analyzed by Elbashbeshy [6]. Rana and Kango [7] discussed the effect of rotation on thermal instability of compressible Walters' (model) elastico-viscous fluid in porous medium. Heat transfer over a stretching surface with variable heat flux in micropolar fluids was presented by Ishak et al. [8]. Chamkha and Aly [9] examined MHD free convective boundary layer flow of a nanofluid along a permeable isothermal vertical plate in the presence of heat source or sink. Thermosolutal convection in Walters' (Model B') elastico-viscous rotating fluid permeated with suspended particles and variable gravity field in porous medium in hydromagnetics was investigated by Rana [10]. Matin et al. [11] presented the MHD mixed convective flow of a nanofluid over a stretching sheet. Chand and and Rana [12] examined the oscillating convection of nanofluid in porous medium. Aziz and Khan [13] studied natural convective flow of a nanofluid over a convectively heated vertical plate. Kuznetsov and Nield [14] analyzed the natural convective flow of a nanofluid past a vertical plate. Khan and Pop [15], investigated the laminar flow of nanofluids past a stretching sheet. Hamad et al. [17] formulated the problem of free convective flow of nanofluid past a semi-infinite vertical plate with influence of magnetic field. Hady et al. [18] investigated the effects of thermal radiation on the viscous flow of a nanofluid and heat transfer over a non-linear sheet. Makinde and Aziz [19] performed the numerical study of boundary layer over a linear stretching sheet. Cheng [20] analyzed the behavior of boundary layer flow over a horizontal cylinder of elliptic cross section in a porous. Narayana and Sibanda [21] elaborated the effects of laminar flow of a nanofluid over an unsteady stretching sheet. Kameswaran et al. [22] investigated flow due to a stretching or shrinking sheet with viscous dissipation and chemical reaction effects. The effects of an unsteady boundary-layer flow and heat transfer of a nanofluid over a porous stretching/shrinking sheet have been investigated by Bachok et al. [23]. Hamad and Ferdows [24] presented the similarity solutions for viscous flow and heat transfer of a nanofluid over a non-linear stretching sheet. The studies on heat generation/absorption effects for boundary layer flow of nanofluids are very limited. Recently, Alsaedi et al. [25] investigated the effects of heat generation/absorption on stagnation point flow of nanofluid over a surface with convective boundary conditions. Thermal instability of Rivlin-Ericksen Elastico-Viscous nanofluid saturated by a porous medium were presented by Chand and Rana [26]. On the onset of thermal convection in rotating nanofluid layer saturating a Darcy-Brinkman porous medium were studied by Chand and Rana [27]. Nandy and Mahapatra [28] examined the effects of slip and heat generation/absorption on MHD stagnation point flow of nanofluid past a stretching/shrinking surface with convective boundary conditions. On the onset of thermosolutal instability in a layer of an Elastico-Viscous nanofluid in porous medium was investigated by Rana et al. [29].

However, to the best of author's knowledge, no attempts have thus far been communicated with regards to free convective boundary layer flow of three-dimensional Oldroyd-B nanofluid over a stretching surface. The aim of the present article is to study the free convective boundary-layer flow of three-dimensional Oldroyd-B nanofluid fluid flow over a stretching sheet. The Oldroyd-B fluid model was employed to describe rheological behavior of viscoelastic nanofluid. The Oldroyd-B fluid model is important because of its applications in the production of plastic sheet and extrusion of polymers through through a slit die in polymer industry. The considered stretched flow problem involves problem involves the significant heat transfer between the sheet and the surrounding fluid. The extrudate in this mechanism starts to solidify as soon as it exits from the die and then sheet is collected by a wind-up roll upon solidification. Physical properties of the cooling medium, e.g., its thermal conductivity has pivotal role in such process. The success of whole operation closely depends upon the viscoelastic character of fluid above the sheet. By applying boundary layer approximations a system of nonlinear partial differential equations is obtained. Then, invoking suitable similarity transformations, we reduced the system into nonlinear ordinary differential equations. This system of coupled nonlinear ordinary differential equations is then solved analytically by using the homotpoy analysis method (HAM). The variations of different flow controlling parameters on the velocity, temperature and concentration profiles are addressed.

## Mathematical Formulation

Consider a steady three-dimensional 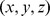 free convective boundary layer flow of an incompressible Oldroyd-B nanofluid over a stretching sheet kept at a constant temperature 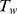 and concentration 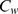. The ambient temperature and concentration far away from the sheet are taken as 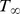 and 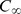, respectively. The flow is due to a bidirectional stretched surface at 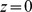. The governing equations for the steady three-dimensional flow of an Oldroyd-B nanofluid, approximated by boundary-layer theory, are [30]

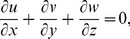
(1)

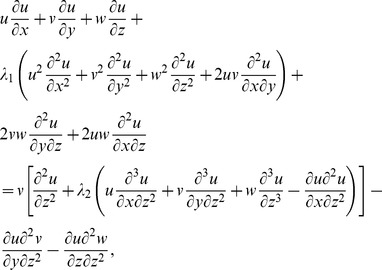
(2)

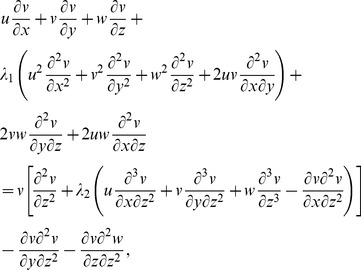
(3)

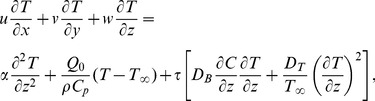
(4)

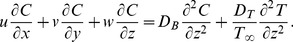
(5)


Here 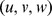 are velocity components, 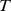 the temperature, 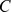 the concentration, 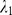 and 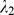 the relaxation and retardation times respectively, 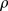 the fluid density, 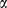 the thermal diffusivity, 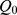 the heat generation/absorption parameter, 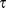 the ratio of effective heat capacity of the nanoparticle material to the heat capacity of the fluid, 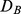 the Brownian diffusion coefficient and 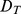 the thermophoresis diffusion coefficient.

Equations (1) to (5) are subjected to the following boundary conditions
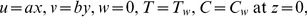
(6)

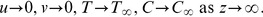
(7)


The similarity variables are introduced as
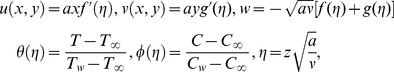
(8)and Eqs.(1)–(7) can be cast as
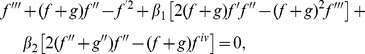
(9)

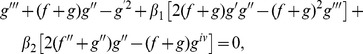
(10)

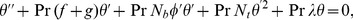
(11)

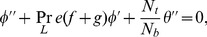
(12)

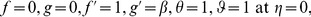
(13)

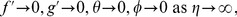
(14)where prime denotes differentiation with respect to 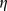. Moreover, 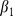 and 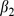 are the Deborah numbers, 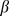 the ratio of stretching rates parameter, 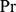 the generalized Prandtl number, 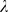 the heat source 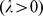 and the heat sink 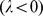 parameter, 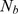 the local Brownian motion parameter, 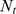 the local thermophoresis parameter and 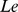 the Lewis number which are defined as
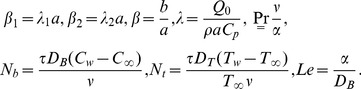
(15)


The physical quantities of interest are the local Nusselt number 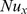 and the local Sherwood number 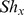, which are defined as
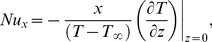
(16)

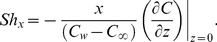
(17)


In terms of dimensionless form one has
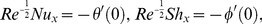
(18)where 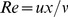 is the local Reynolds number.

## Convergence of the Homotopy Solutions

The problems containing non-linear coupled ordinary differential equations (19)–(12) subjected to boundary conditions (13)–(14) have been computed analytically by the homotopy analysis method (HAM). In the HAM role of the auxiliary parameters *ħ_f_*, *ħ_g_*, *ħ_θ_*, and *ħ_φ_* is of key importance because they control the convergence of the series solution. The most suitable value of these auxiliary parameters is calculated by considering minimum square error which is given by
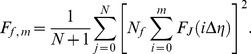
(19)


Table 1 ensure the convergence of the series solution which shows that the convergent solution for the velocity is obtained at 20th-order of approximation whereas such a convergence for temperature and concentration is achieved at 26th-order of approximation.

**Table 1:** Convergence of the homotopy solutions when 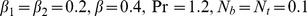 and 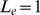 are fixed.

Order of approximation	−f ′′(0)	−g′′(0)	−θ′(0)	−φ′(0)
1	1.00420	0.337840	0.622000	0.352000
5	1.02196	0.328912	0.549080	0.493576
10	1.02155	0.328848	0.549423	0.489147
15	1.02154	0.328870	0.549446	0.488882
20	1.02154	0.328869	0.549438	0.488934
26	1.02154	0.328869	0.549438	0.488939
30	1.02154	0.328869	0.549438	0.488939
35	1.02154	0.328869	0.549438	0.488939

## Numerical Results and Discussion

The aim of this section is to analyze the influence of the various physical parameters on the velocity, temperature and nanoparticle fields respectively. Figs. 1–14 are plotted to see the variation of the Deborah numbers 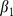 and 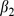, Prandtl number Pr, heat source 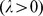 or sink 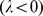 parameter, Brownian motion parameter 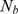 and thermophoresis parameter 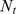 on the fluid temperature and concentration fields.

**Figure 1:**
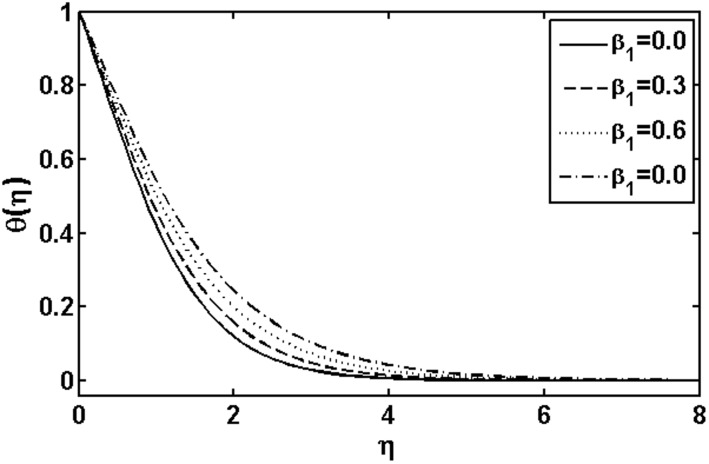
Variation of 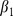 on 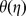 when 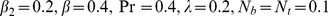 and 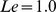 are fixed.

**Figure 2:**
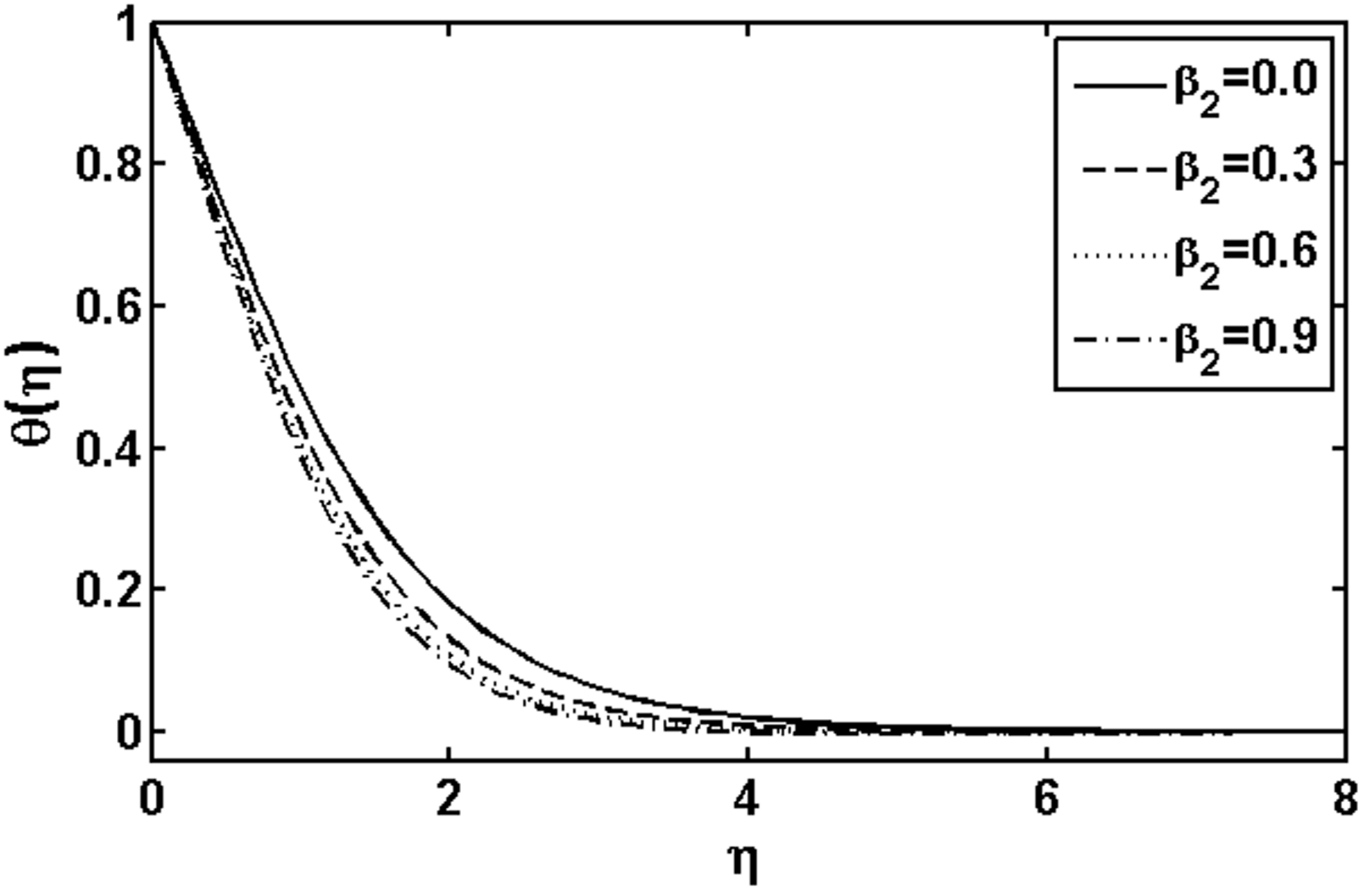
Variation of 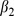 on 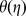 when 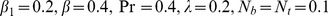 and 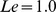 are fixed.

**Figure 3:**
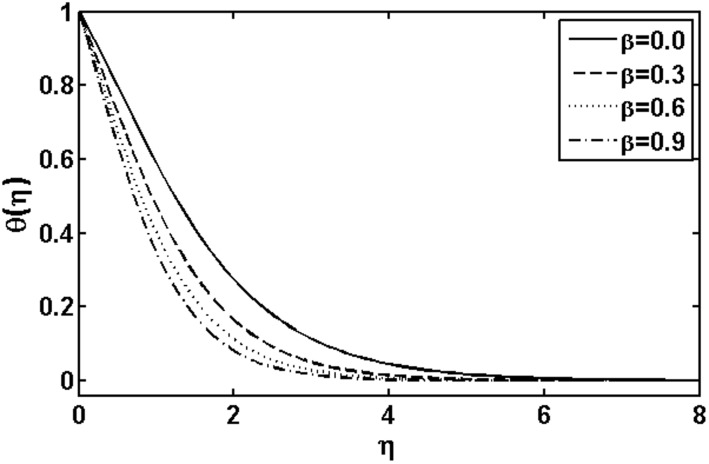
Variation of 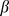 on 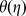 when 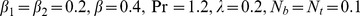 and 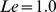 are fixed.

**Figure 4:**
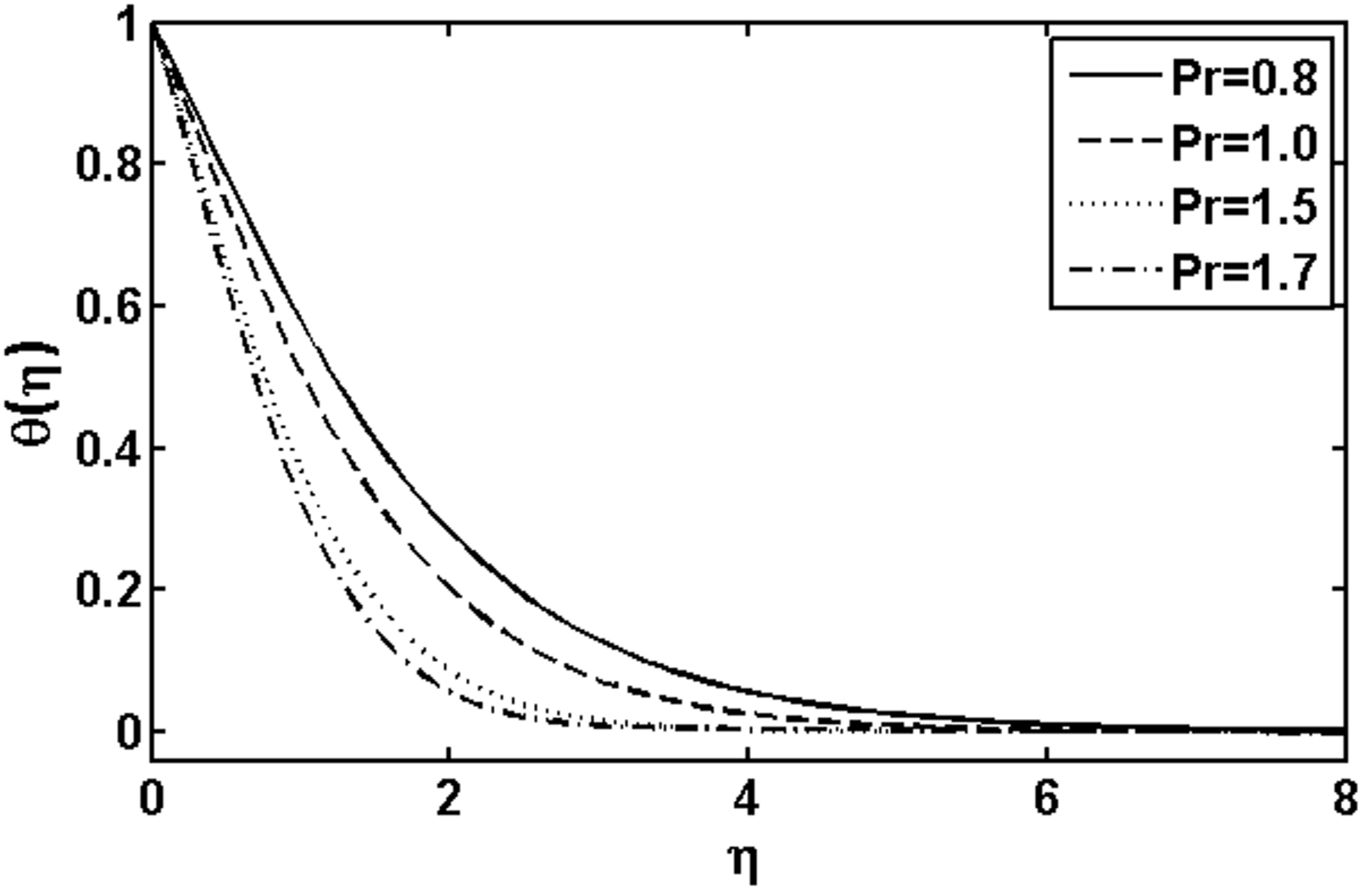
Variation of 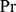 on 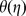 when 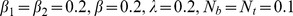 and 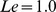 are fixed.

**Figure 5:**
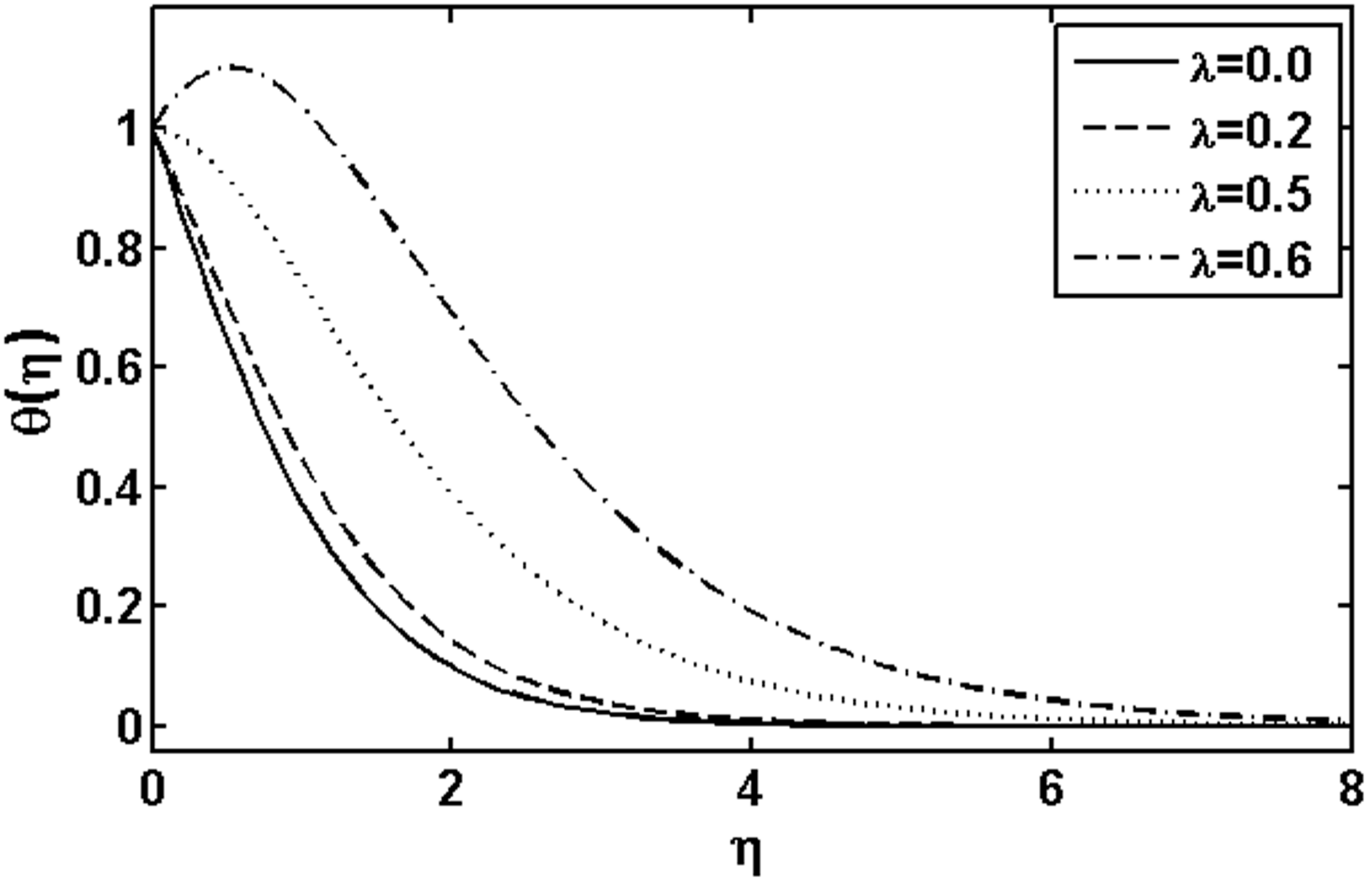
Variation of 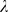 on 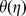 when 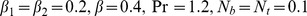 and 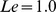 are fixed.

**Figure 6:**
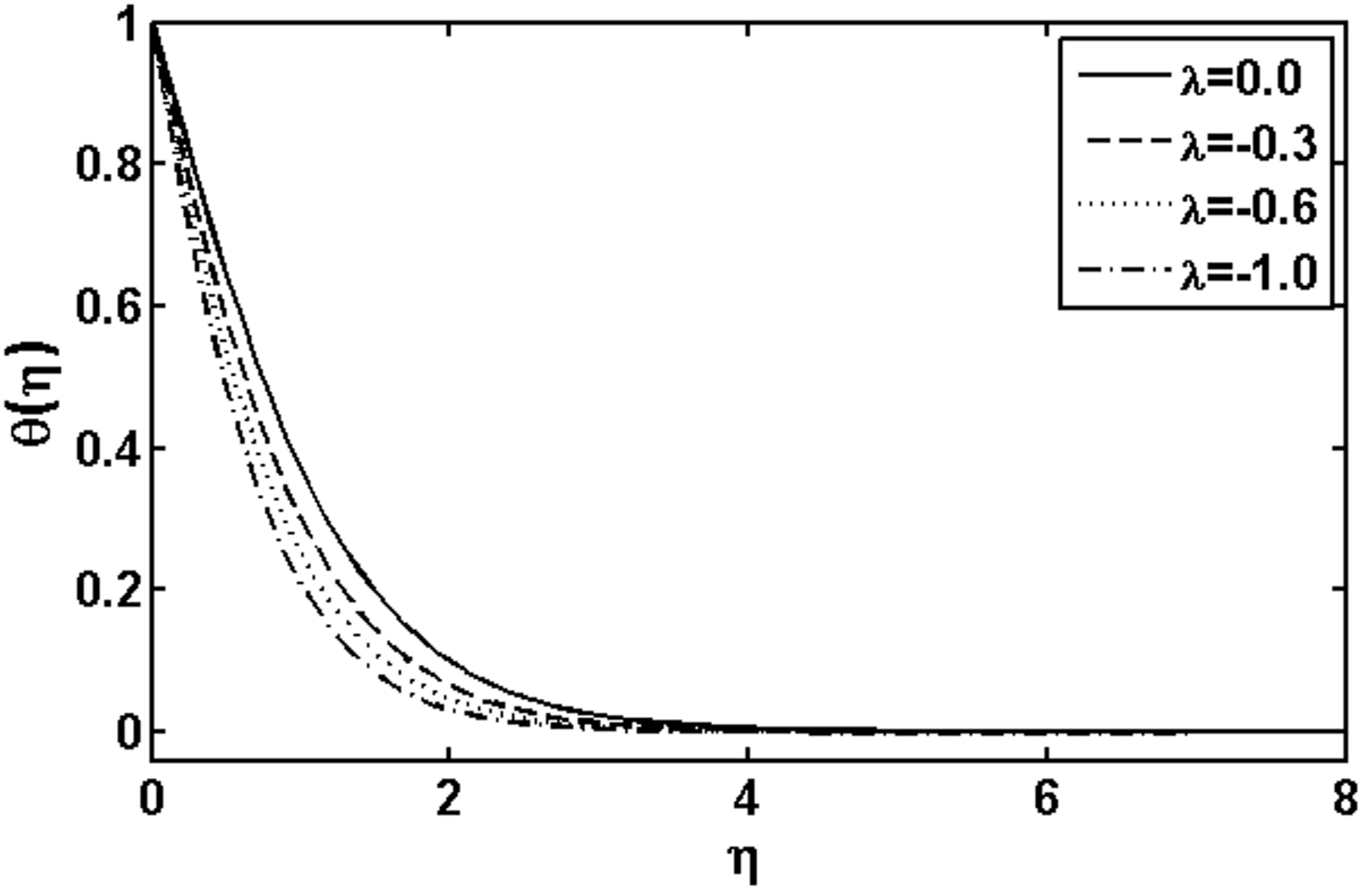
Variation of 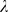 on 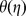 when 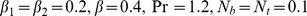 and 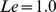 are fixed.

**Figure 7:**
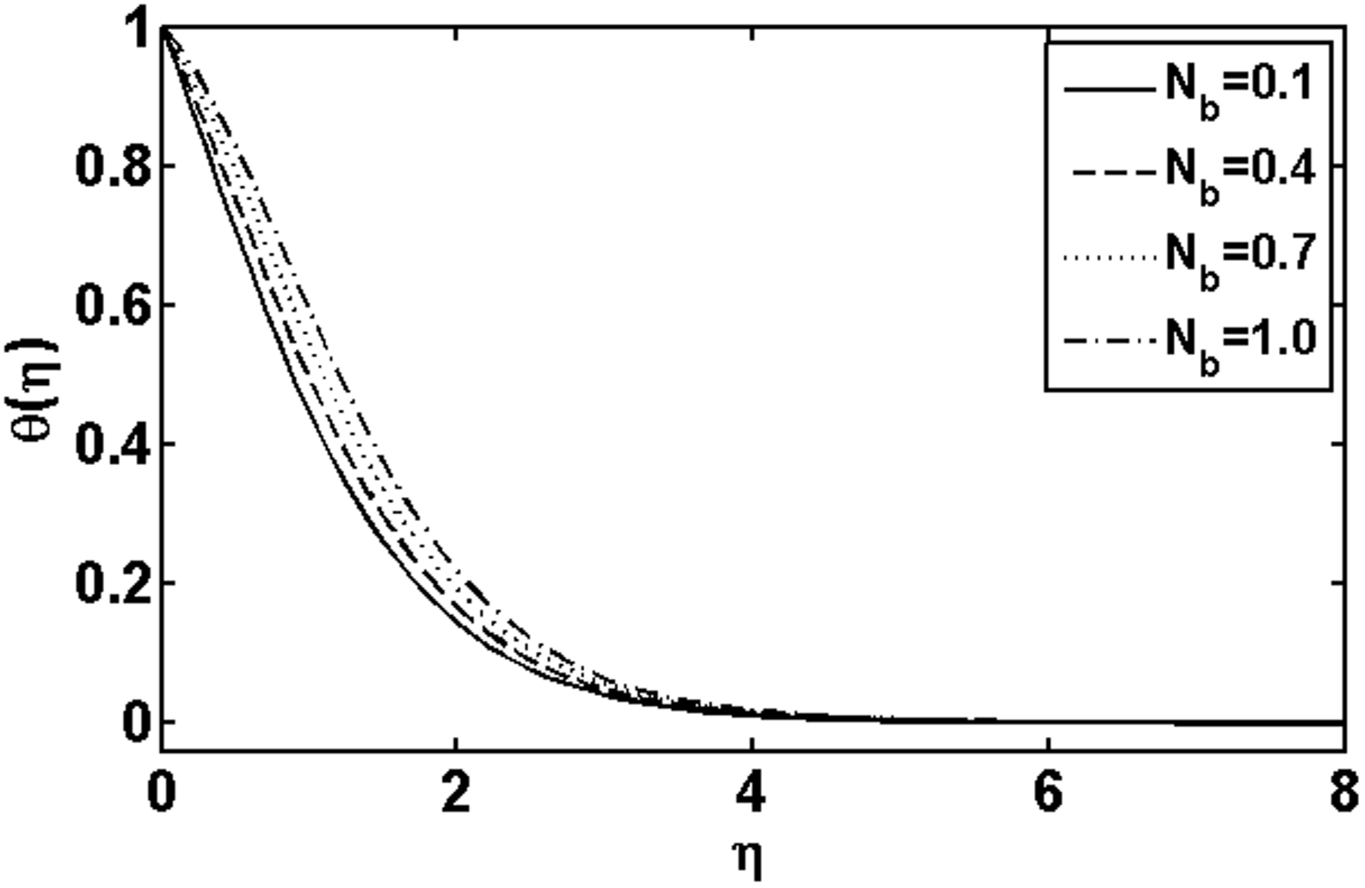
Variation of 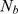 on 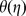 when 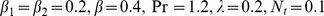 and 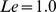 are fixed.

**Figure 8:**
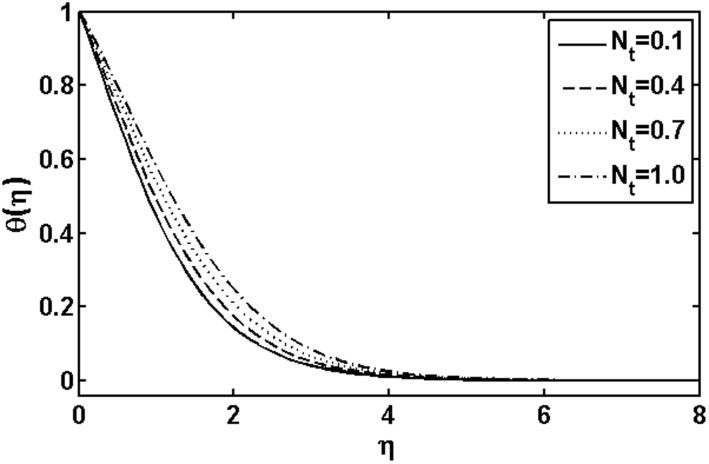
Variation of 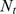 on 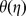 when 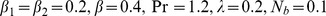 and 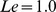 are fixed.

**Figure 9:**
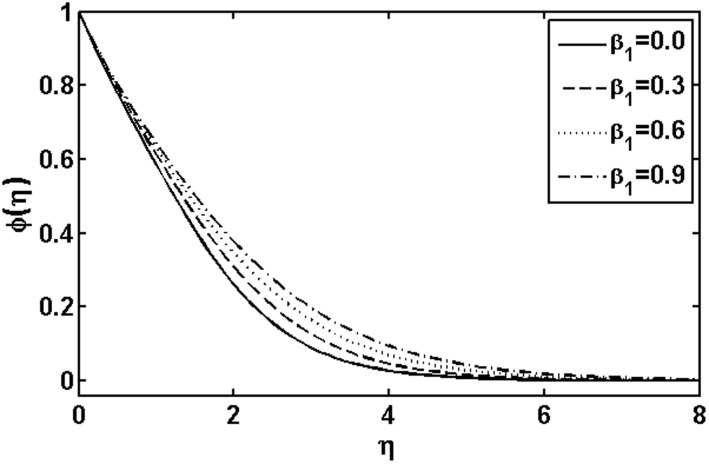
Variation of 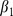 on 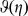 when 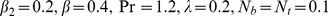 and 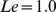 are fixed.

**Figure 10:**
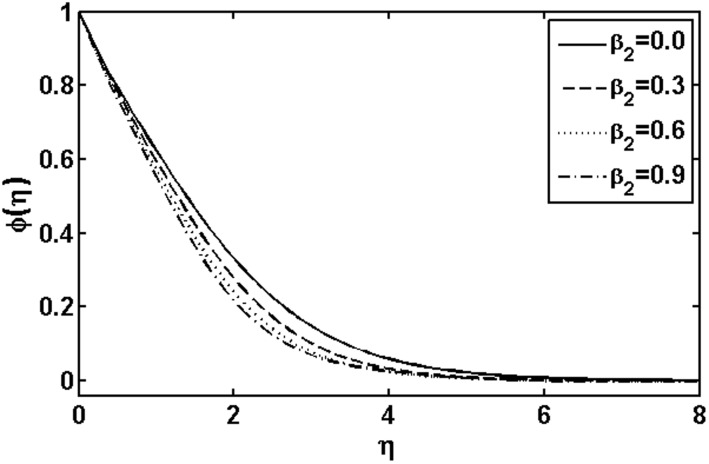
Variation of 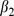 on 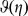 when 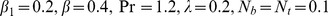 and 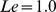 are fixed.

**Figure 11:**
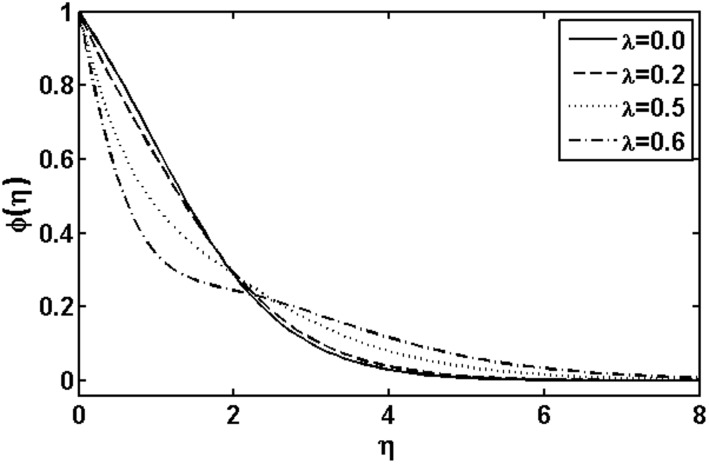
Variation of 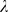 on 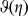 when 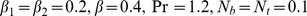 and 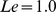 are fixed.

**Figure 12:**
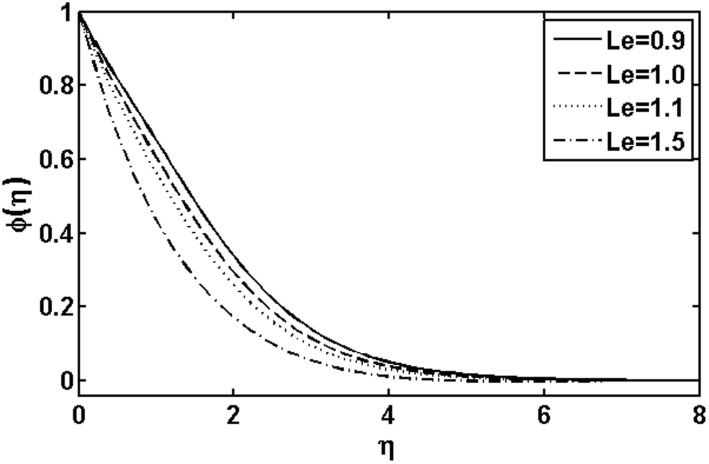
Variation of 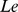 on 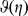 when 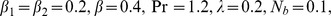 and 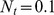 are fixed.

**Figure 13:**
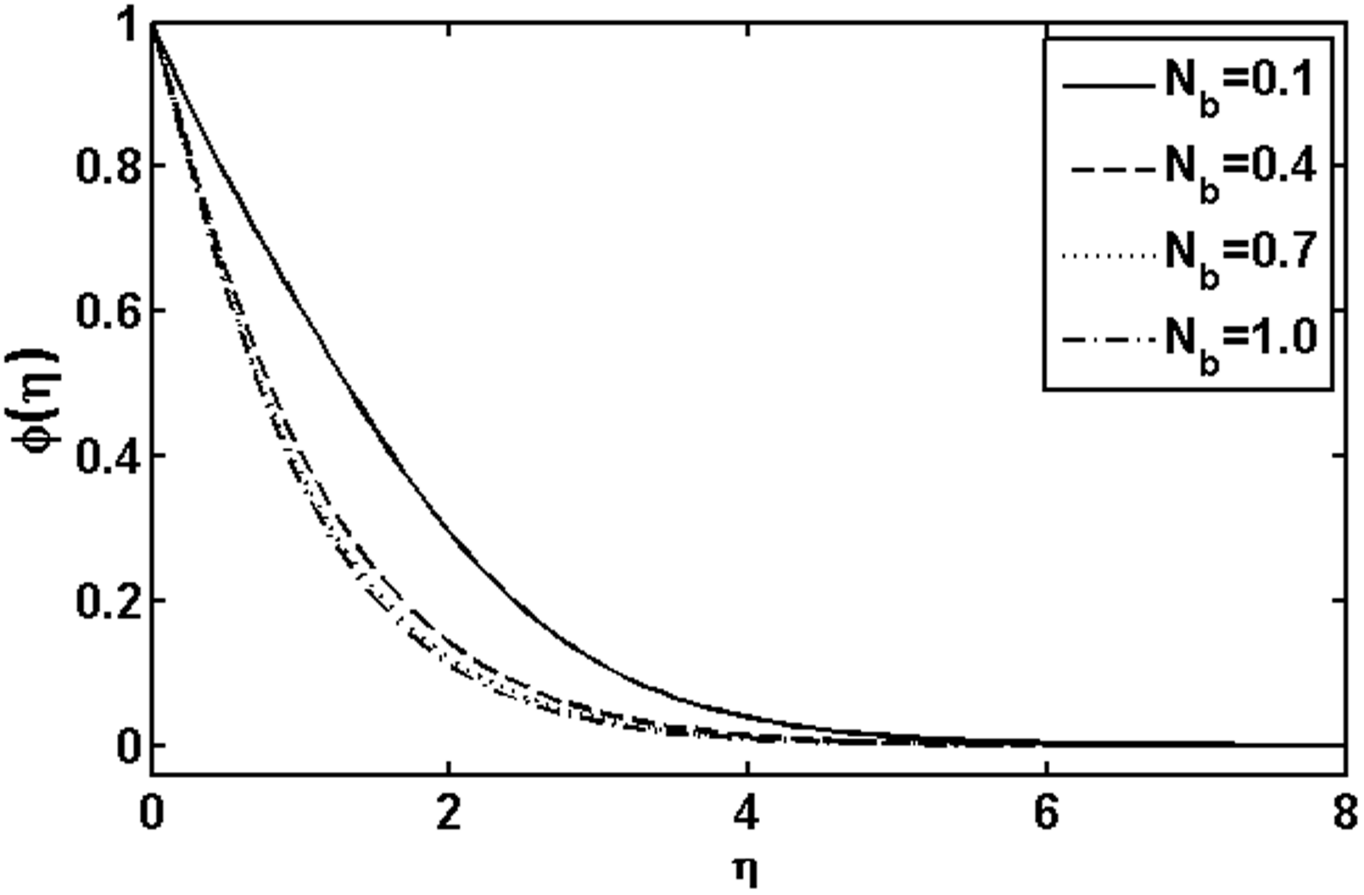
Variation of 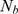 on 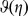 when 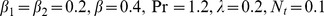 and 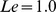 are fixed.

**Figure 14:**
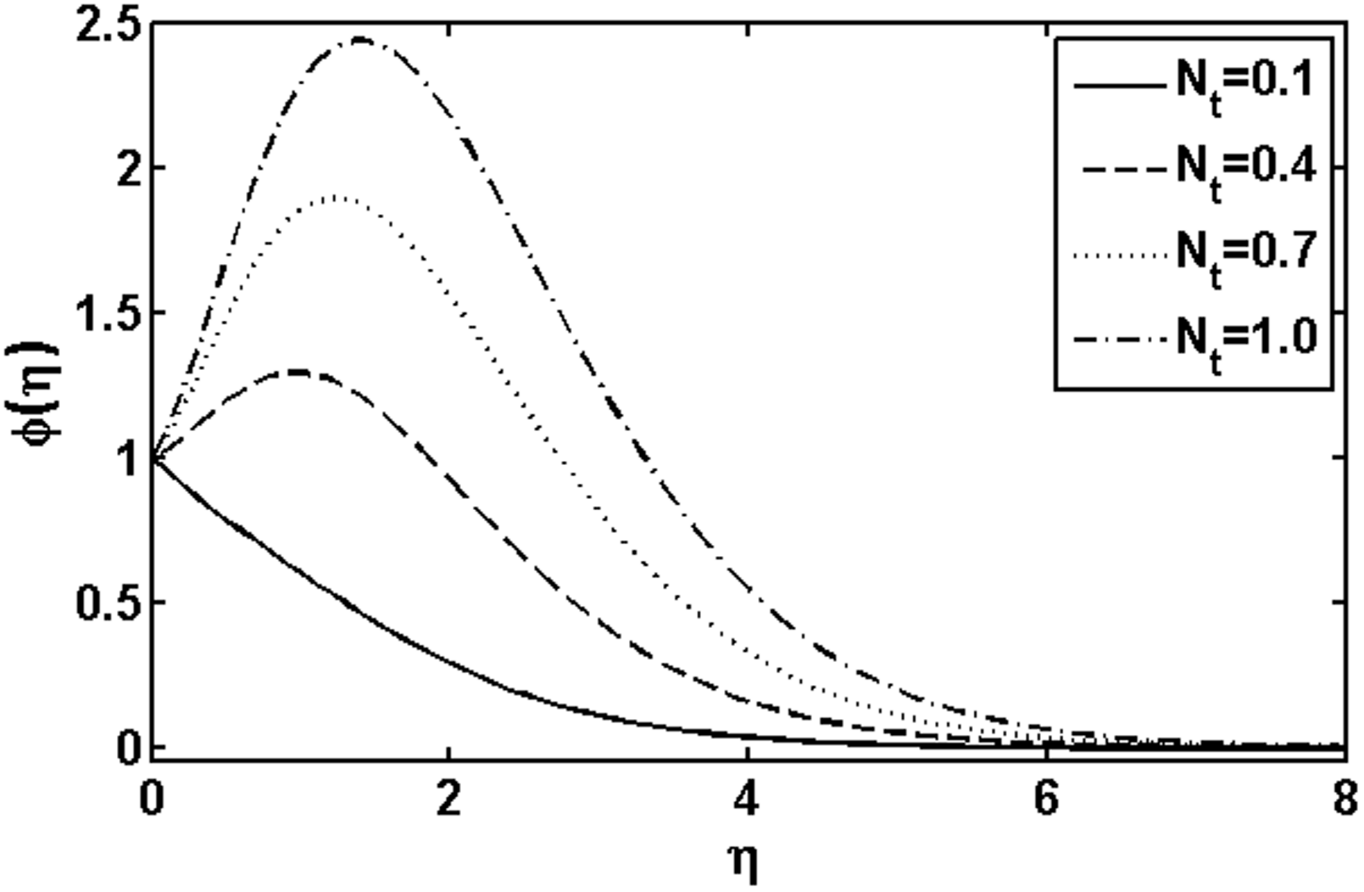
Variation of 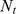 on 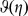 when 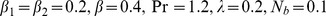 and 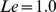 are fixed.

Fig. 1 shows the influence of the Deborah number 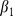 on the temperature field. By increasing Deborah number 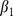 both the fluid temperature and thermal boundary layer thickness increases. This is due to fact that the Deborah number 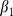 involves relaxation time 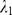. An increase in the relaxation time leads to increase in the temperature and boundary layer thickness. Fig. 2 illustrates the effects of the Deborah number 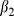 on the temperature field. From this figure, it is noted that the behavior of the Deborah number 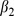 is opposite to that of 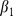. This is due to fact that the retardation time provides resistance which causes a reduction in the temperature and thermal boundary layer thickness. Fig. 3 presents the effects of the stretching parameter 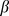 on the fluid temperature 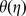. We observed that the temperature and thermal boundary layer thickness reduce with the increasing 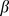. Fig. 4 illustrates the influence of the Prandtl number 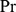 on the temperature field. We observed that the temperature and thermal boundary layer thickness are reduced for large Prandtl number. Since thermal diffusivity is an agent which plays a key role for lower or higher temperature. Hence resulting larger value of the Prandtl number corresponds to diminishing of the thermal diffusivity resulting in a temperature decrease. Figs. 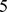 and 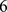 are plotted to analyze the effects of the heat source parameter 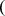 when 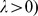 and heat sink parameter 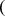 when 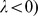, respectively. It is observed that temperature of the fluid increases with the increase in the heat source parameter and an opposite behavior is observed for heat sink parameter. The influence of the Brownian motion parameter 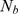 and thermophoresis parameter 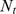 on the temperature is depicted through Figs. 7 and 8, respectively. It is observed that the temperature and thermal boundary layer thickness increases as the Brownian motion parameter 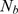 increases. Physically, this is due to the fact that with an increase of the Brownian motion parameter 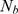 the random motion of particle increases which results in an enhancement in the temperature profile. The temperature and thermal boundary layer thickness are detected to increase with an increase in thermophoresis parameter 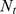 (Fig. 8). In fact with the increase of the thermophoresis parameter 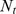 the difference between the wall temperature and reference temperature increases which causes increase in temperature profile.

In Figs. 9 and 10, we plotted the concentration profile for various values of the Deborah numbers 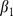 and 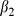, respectively. As the Deborah numbers 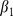 increases, the concentration profile as well as concentration boundary layer thickness increase. However, the effects of 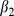 on the concentration profile are quite opposite to that of 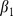. Fig. 11 shows the influence of the heat generation parameter 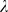 on the concentration profile. A decrease in the concentration profile and concentration boundary layer thickness near the plate is noted while the reverse effect is reported far away from the plate with the increasing value of the heat generation parameter 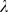. Fig. 12 illustrates the influence of the Lewis number 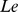 on the concentration profile 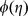. It is noted that the concentration profile increases by increasing the Lewis number 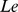 as Lewis number is inversely proportional to the diffusion coefficient. Thus an increase in Lewis number yields a decrease in diffusion which finally results in a decrease of mass fraction function 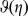. The variations with 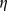 of the concentration profile for different values of the Brownian motion parameter 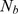 and thermophoresis parameter 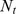 are presented in Figs. 13 and 14, respectively. In Fig. 13, it is observed that concentration profile increases with the increasing of the Brownian motion parameter 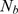. This is due to the dependency of the concentration on the temperature field and we expect that a lower Brownian motion parameter allow a deeper penetration of the concentration. On the other hand, a qualitatively opposite trend in the concentration profile is observed as the thermophoresis parameter 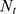 increases. Further, it is noticed that the thermophoresis parameter 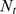 affects the concentration profile more than Brownian motion parameter 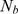 does.

Numerical values for the velocity gradients 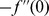 and 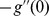 are compared with the existing literature in the absence of both nanoparticles and non-Newtonian effects and shown in table 2, where they are found to be in excellent agreement, cementing the validity of the present results. Table 3 gives comparison of local Nusselt number −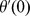 with the results obtained by Khan and Pop [15] and Nadeem and Hussain [32] Table 4 provides comparison of local Nusselt number −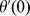 and local Sherwood number −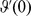 for different values of the Brownian motion parameter 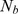 and the thermophoresis parameter 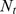 with existing results obtained by Nadeem 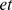 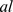.[33]. Table 5 is prepared for the variation of the local Nusselt number (heat transfer rate) and the local Sherwood number (concentration rate) for different values of the involved parameters. It is reported that the local Nusselt number −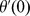 increases when 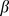 and 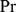 increase whereas it decreases as 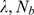, 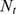 and 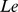 increase. It is evident from table 5 that the local Sherwood number −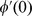 increases with the increase of the parameters 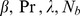 and 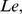 however, it decreases with the increase of 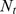.

**Table 2:** A comparison for the velocity gradients for different values of 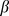 when 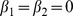 are fixed.

β	HPM result [31]	HPM result [31]	Exact result [31]	Exact result [31]	Present result	Present result
	−f′′(0)	−g′′(0)	−f′′(0)	−g′′(0)	−f′′(0)	−g′′(0)
0.0	1.0	0.0	1.0	0.0	1.0	0.0
0.1	1.02025	0.06684	1.020259	0.066847	1.02026	0.06685
0.2	1.03949	0.14873	1.039495	0.148736	1.03949	0.14874
0.3	1.05795	0.24335	1.05794	0.243359	1.05795	0.24336
0.4	1.07578	0.34920	1.075788	0.349208	1.07578	0.34921
0.5	1.09309	0.46520	1.093095	0.465204	1.09309	0.46521
0.6	1.10994	0.59052	1.109946	0.590528	1.10994	0.59053
0.7	1.12639	0.72453	1.126397	0.724531	1.12639	0.72453
0.8	1.14248	0.86668	1.142488	0.866682	1.14249	0.86668
0.9	1.15825	1.01653	1.158253	1.016538	1.15826	1.016538
1.0	1.17372	1.17372	1.173720	1.173720	1.17372	1.17372

**Table 3:** Comparision of results for the local Nusselt number −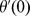 in the absence of non-Newtonian parameters and nanoparicles when 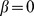 with the work of Khan and Pop [15] and Nadeem and Hussain [32]

Pr	Present result	Khan and Pop [15]	Nadeem and Hussain [32]
0.07	0.066	0.066	0.066
0.20	0.169	0.169	0.169
0.70	0.454	0.454	0.454
2.0	0.911	0.911	0.911

**Table 4:** Comparision of results for the local Nusselt number −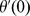 and local Sherwood number −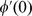 in the presence of nanoparticle of when 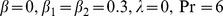 and 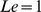 are fixed with the work of Nadeem et al. [33].

N_t_	N_b_	Present result	Present result	Nadeem et al. [33]	Nadeem et al. [33]
		−θ′(0)	φ′(0)	−θ′(0)	φ′(0)
0.3	0.3	0.33984	1.83994	0.33988	1.83935
0.5	0.3	0.24088	1.95813	0.24099	1.95862
0.3	0.5	0.14814	1.87029	0.14820	1.87035
0.5	0.5	0.10478	1.94565	0.10486	1.94572

**Table 5:** Variations of the Local Nusselt number and local Sherwood number with 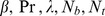 and 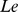 when 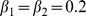 are fixed.

β	Pr	λ	N_b_	N_t_	Le	−θ′(0)	φ′(0)
0.0	1.2	0.2	0.1	0.1	1.0	0.351853	0.474210
0.3						0.509837	0.482838
0.4						0.549438	0.488939
0.5	1.0					0.513728	0.430930
	1.1					0.551238	0.463216
	1.3					0.617393	0.527723
		0.0				0.757208	0.356670
		0.1				0.676907	0.422143
		0.4				0.335390	0.690221
			0.2			0.540514	0.683362
			0.3			0.497586	0.745281
			0.4			0.456841	0.775726
				0.2		0.575795	0.199343
				0.4		0.505394	−0.277762
				0.5		0.480757	−0.464410
					0.8	0.591165	0.352548
					0.9	0.588200	0.426414
					1.1	0.583427	0.560578

## Concluding Remarks

This study has analyzed the effects of the heat generation/absorption on three-dimensional flow of an Oldroyed-B nanofluid over a bidirectional stretching sheet. From the present investigation, the main observations were as follows:

- Qualitatively, effects of the Deborah numbers 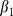 and 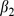 on the temperature and concentration profiles were similar.
- The temperature profile as well as thermal boundary layer thickness were increased by increasing both the Brownian motion parameter 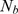 and thermophoresis parameter 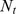.
- The temperature of the fluid and thermal boundary layer thickness is enhanced when there is a increase in the heat generation parameter 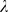.
- The concentration profile was decreased with the increase of the Brownian motion parameter 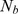 and a quite opposite behavior was noted with increasing thermophoresis parameter 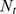.
- It was noted that the thermophoresis parameter 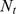 affected the concentration profile more than the Brownian motion parameter 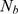 did.
- An increase in the heat generation parameter 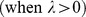 corresponds to reduction in the values of the local Nusselt number −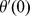 while the opposite behavior is observed for the local Sherwood number −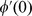.
- The magnitude of the local the local Nusselt number −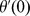 decreases with the increase of the Brownian motion parameter 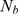.
- The magnitude of the local the local Sherwood number −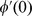 increase with the increase of the Brownian motion parameter 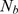.
